# Psychosomatic Status, Personality Traits, and Coping Styles of Bereaved and Non-Bereaved Survivors of the 2008 Wenchuan Earthquake, China

**DOI:** 10.3389/fpsyt.2016.00017

**Published:** 2016-03-11

**Authors:** Yan-hui Xiang, Xinli Chi, Yi-qi Jiang, Rui-fang Wang, Lei Mo

**Affiliations:** ^1^Center for Study of Applied Psychology, South China Normal University, Guangzhou, Guangdong, China; ^2^College of Psychology and Sociology, Shenzhen University, Shenzhen, Guangdong, China; ^3^School of Psychology, South China Normal University, Guangzhou, Guangdong, China

**Keywords:** personality, coping, psychosomatic status, bereaved survivors, Wenchuan earthquake

## Abstract

**Objectives:**

This study examined personality, coping styles, and psychosomatic characteristics and their relationships in bereaved and non-bereaved earthquake survivors.

**Study design:**

Cross-sectional survey.

**Methods:**

A survey was conducted with a sample of 102 non-bereaved survivors and 79 bereaved survivors from Mianyang, Anyang, and similar districts 2 weeks after Wenchuan earthquake. Survivors completed questionnaires, including items about demographics, personality characteristics, coping styles, and psychosomatic status.

**Results:**

Bereaved survivors had lower scores for gregariousness, trust, and optimism, but higher scores for depressed mood, loneliness, becoming easily fearful, irritation, and anxiety than non-bereaved survivors. In addition, bereaved participants scored higher for avoiding problems, self-blame, and fantasy coping styles than non-bereaved ones. Personality and coping styles significantly correlated with psychosomatic status in bereaved and non-bereaved survivors. Optimism and openness to feelings personality characteristics, and self-blame, avoiding problems, and rationalization coping styles significantly predicted psychosomatic status of bereaved survivors, whereas openness to fantasy, optimism, order, and trust personality characteristics, and self-blame and avoiding problems coping styles significantly predicted psychosomatic status of non-bereaved survivors.

**Conclusion:**

Earthquake survivors experienced post-traumatic stress disorder (PTSD) symptoms and negative emotions. Bereaved survivors experienced more serious PTSD symptoms and negative emotions relative to non-bereaved survivors. Appropriate psychological crisis interventions should be conducted for earthquake survivors, especially bereaved survivors.

## Introduction

Natural disasters, such as earthquakes, can cause considerable bodily injury, fear, and property damage, which may result in psychological injury (1, 2), including psychological trauma [i.e., post-traumatic stress disorder (PTSD) symptoms] and negative emotions (i.e., depression) (3, 4). A large number of studies have examined various psychological factors (e.g., psychosocial competencies, life stress, and previous psychiatric illness) that were correlated with PTSD symptoms and negative emotions of earthquake survivors (5–7). Among these factors, personality and coping styles are of interest.

Personality is defined as consistent patterns of behavior reflecting thoughts and emotions, and it is considered stable or slow changing. Previous research about the relationship between personality and traumatic events has typically regarded personality as an independent variable predicting the incidence of PTSD or related negative emotions. For example, an investigation of Australian Vietnam veterans found that having neurotic traits was significantly and positively correlated with combat-related PTSD (8). A study of survivors of fire found that neurotic traits formed an effective index for predicting negative emotions of disaster survivors (9). Another study also found that personality characteristics could predict PTSD symptoms and related adverse emotions in earthquake survivors (10). Thus, personality characteristics may be significant predictors of PTSD symptoms and negative emotions in disaster survivors.

Coping styles refer to active efforts through positive or negative behaviors to deal with stress, involving a series of cognitive and behavioral strategies (11). Positive coping can help to ameliorate adverse symptomatology, whereas avoidant coping can strengthen negative emotional responses to earthquakes (12, 13). A large number of studies have found that coping is strongly related to emotions or PTSD symptoms after trauma. For example, one study examining psychological distress and coping strategies subsequent to the 1995 Dinar earthquake in Turkey found that the use of coping styles, such as the problem solving/optimistic approach, was negatively related to distress levels (14). Another study found that the number of coping strategies (e.g., problem-focused coping and active and avoidant emotional coping) significantly positively correlated with PTSD severity and complicated grief among American college students experiencing the unexpected death of an immediate family member and close friend (15). These findings were also confirmed by studies on the Indian Ocean tsunami, hurricane, and earthquake (13, 16, 17). These studies revealed that coping styles could predict PTSD and negative emotions (12).

In short, prior research has provided valuable information on personality, coping styles, and psychosomatic status in trauma events. However, research gaps still exist. As mentioned above, disaster survivors may face tremendous mental anguish due to bodily injury, fear, or property damage, but the most profound may be the loss of family members or relatives in a disaster (18). Previous studies have found that, compared with non-bereaved survivors, bereaved survivors may experience greater psychological trauma (i.e., PTSD symptoms) and negative emotions (i.e., depression) (3, 4, 19–21). However, few studies have compared personality and coping styles between bereaved survivors and non-bereaved survivors in China (19). Additionally, to the best of our knowledge, no research has examined whether the personality traits and coping styles predicting psychosomatic status differ between bereaved and non-bereaved survivors. Examining personality characteristics, coping styles, and psychosomatic status shortly after trauma can provide useful information for the development of appropriate emergency interventions for disaster survivors.

Against this background, the present study was designed to address two primary research questions such as (a) what were the differences between bereaved and non-bereaved earthquake survivors in terms of personality, coping styles, and psychosomatic status? and (b) what were the factors of personality traits and coping styles associated with psychosomatic status in the two groups?

## Materials and Methods

### Participants

A convenience sample was drawn of 210 people from earthquake disaster areas; 187 returned their questionnaires (Beichuan, 71; An county, 76; Qu county, 22; Mianyang and other areas, 18). Twenty-three survivors failed to complete the questionnaires for physical or emotional reasons, and 181 indicated whether they had lost relatives in the earthquake. The final sample consisted of 102 survivors who had not lost relatives and 79 survivors who had lost relatives.

### Procedure

A week after the Wenchuan Earthquake, the Chinese government set up temporary housing in Mianyang Jiuzhou Gymnasium, South Lake Gymnasium, and Southwest University of Finance, where they provided basic living supplies to survivors. The Red Cross Society of China organized a group of experts in order to collect information on the psychological stress of survivors. The authors of this manuscript were among these experts. Before the survey, all researchers were trained in the study procedure. In the temporary housing are the researchers invited survivors to respond a survey and inform them the survey intention. If they were willing to participate, the survey would continue, if not, the survey would give up. Because we believed that survivors would be experiencing a highly diverse range of extreme emotions, and because we were involved in providing psychological intervention for survivors participating in the questionnaire survey, we administered the survey only after determining the individual’s emotional state and willingness. Among the survivors, 12 survivors had different levels of education. These survivors (e.g., illiteracy) gave oral responses to the questionnaire, while others filled it independently. Those who completed the questionnaires were offered compensation in the form of a radio.

### Measures

#### Big Five Personality Traits

Personality traits were assessed using the Revised NEO Personality Inventory, a measure of the Big Five personality traits. This 240-item tool was developed by Costa and McCrae (22). It has been translated into Chinese and has been widely used in China; the Chinese version has good reliability and validity (23, 24). Considering the disaster conditions and survivors’ emotions, we adopted an abbreviated version measuring only 15 specific personality traits (e.g., gregariousness, warmth, dutifulness, and trust), following previous studies. The abbreviated questionnaire consisted of 89 items rated on a 5-point scale from 1 (“totally disagree”) to 5 (“totally agree”). Previous studies reported a Cronbach’s alpha of 0.86 for the abbreviated version (25, 26). In the present study, Cronbach’s alpha of the subscales for various personality traits ranged from 0.71 to 0.89. Thus, the scale had adequate internal consistency.

#### Coping Style Questionnaire

The 62-item Coping Style Questionnaire (10), which measures the dimensions of avoiding problems, fantasizing, self-blaming, asking for help, rationalization, and problem solving, was adopted to examine the coping styles of survivors in the study. The measure has been widely used in the previous research. Questions were answered on a 5-point scale ranging from 1 (“not sure at all”) to 5 (“very sure”). A compound score for each coping style was calculated by averaging the scores of all the items for that coping style. In the study by Zhang et al. (10), Cronbach’s alpha was 0.88 and the split-half reliability coefficient was 0.87. In the present study, Cronbach’s alpha and the split-half reliability coefficient were 0.85 and 0.83, respectively.

#### Psychosomatic Situation Scale

The Psychosomatic Situation Scale was employed to investigate the psychosomatic status of survivors (10). The scale was initially developed for assessing Chinese children’s mental health (27) and subsequently modified by Zhang et al. (10) to assess individuals’ earthquake-related post-traumatic mental health. The modified scale contains 53 items and 3 subscales: emotion (depression, loneliness, irritability, anger, anxiety, and fear), cognitive dysfunction (distraction and inefficacy), and physiological adaptation (disinterest insomnia, early gets tired, and decreased appetite). Each item was answered on a 5-point scale ranging from 1 (“no experience”) to 5 (“extremely severe experience of the state”). Zhang et al. (10) reported a Cronbach’s alpha of 0.89 in her study; in this study, Cronbach’s alpha was 0.92.

### Statistical Analysis

Chi-square tests and an independent samples *t*-test were conducted to examine differences in demographic variables, personality, coping styles, and psychosomatic status. Correlation analysis was carried out to examine the relationship between personality, coping styles, and psychosomatic situation. Regression analysis was used to explore the predicting effects of personality and coping styles on psychosomatic status. All analyses were performed using SPSS 19.0 (IBM Corp., Armonk, NY, USA).

## Results

### Profiles of Participants

The overall average age of participants was 39 ± 1 years. Except for education level (χ^2^ = 17.59, *P* = 0.001), demographic characteristics (age, gender, marriage status, surround in ruins, and see body) did not significantly differ between bereaved and non-bereaved survivors (Table 1).

**Table 1:** **Sample characteristics in bereaved and non-bereaved survivors**.

	Bereaved survivors	Non-bereaved survivors	Significance test of difference
Age	41.05 + 14.43	36.37 + 11.50	*t* = 2.358, *P* = 0.055
Sex			χ^2^ = 0.031, *P* = 0.859
Male	53 (52.0%)	40 (50.6%)	
Female	49 (48.0%)	39 (49.4%)	
Marriage status			χ^2^ = 0.438, *P* = 0.803
Single	16 (15.7%)	14 (18.2%)	
Married	86 (84.3%)	63 (81.8%)	
Education level			χ^2^ = 24.495, *P* < 0.001
Primary school or less	19 (24.4%)	16 (22.2%)	
Junior middle school	13 (14.0%)	31 (43.1%)	
High school	23 (24.7%)	16 (22.2%)	
College or higher	38 (40.9%)	9 (12.5%)	
Surround in ruins			χ^2^ = 1.329, *P* = 0.249
Yes	66 (67.3%)	58 (75.3%)	
No	32 (32.7%)	19 (24.7%)	
See body			χ^2^ = 0.012, *P* = 0.913
Yes	41 (46.6%)	37 (47.4%)	
No	47 (53.4%)	41 (52.6%)	

### Psychosomatic Status

As shown in Table 2, bereaved participants had significantly higher scores than non-bereaved participants on the following variables: depressed mood, loneliness, becoming easily fearful, irritation, anxiety, becoming easily angry, compulsive re-experiencing, escape, suicide, guilt, indifference, distraction, disinterest, insomnia, decreased appetite, and becoming tired easily (*p*’s < 0.05). There was no significant difference in coping efficacy between bereaved and non-bereaved participants.

**Table 2:** **Psychosomatic status differences between bereaved and non-bereaved survivors (*M* ± SD)**.

Factor	Bereaved survivors	Non-bereaved survivors	*t*
Depressed mood	3.18 ± 1.01	2.49 ± 0.95	−4.69**
Felt lonely	2.97 ± 1.01	2.36 ± 0.91	−4.21**
Easily fearful	4.04 ± 0.85	3.41 ± 1.18	−4.04**
Irritation	3.39 ± 0.89	2.58 ± 1.00	−5.66**
Anxiety	3.60 ± 0.90	3.01 ± 1.10	−3.85**
Easily angry	2.90 ± 0.99	2.31 ± 0.98	−4.00**
Compulsive re-experiencing	3.59 ± 0.98	2.81 ± 1.14	−4.88**
Escape anything	2.92 ± 0.93	2.61 ± 0.92	−2.21*
Suicide	2.57 ± 1.07	2.02 ± 0.94	−3.65**
Felt guilty	3.33 ± 1.15	2.45 ± 1.10	−5.24**
Indifferent to anything	3.33 ± 1.06	2.67 ± 0.99	−4.33**
Distraction	3.08 ± 1.00	2.66 ± 1.10	−2.67**
Disinterest	2.63 ± 1.47	2.01 ± 1.16	−3.19**
Inefficacy	3.40 ± 0.84	3.61 ± 0.77	1.76
Insomnia	3.72 ± 1.01	3.07 ± 1.22	−3.80**
Decreased appetite	3.05 ± 1.06	2.47 ± 1.00	−3.75**
Easily gets tired	3.39 ± 1.01	3.05 ± 1.08	−2.14*

*P < 0.05.

**P < 0.01.

*The tests used for assessment of the respective psychological variables*.

### Personality Characteristics

As can be seen in Table 3, bereaved survivors reported significantly lower gregariousness, trust, and optimism than non-bereaved survivors (*p*’s < 0.05). The remaining personality traits did not significantly differ between bereaved and non-bereaved participants.

**Table 3:** **Personality differences between bereaved and non-bereaved survivors (*M* ± SD)**.

Factor	Bereaved survivors	Non-bereaved survivors	*t*
Gregariousness	4.11 ± 0.55	3.85 ± 0.80	2.53*
Openness to fantasy	2.96 ± 0.67	3.10 ± 0.68	−1.36
Excitement seeking	3.38 ± 0.57	3.47 ± 0.63	−1.02
Trust	3.90 ± 0.61	3.69 ± 0.54	2.40*
Altruism	3.72 ± 0.45	3.69 ± 0.44	0.51
Compliance	7.70 ± 0.62	3.53 ± 0.70	1.68
Tender-mindedness	3.94 ± 0.66	3.96 ± 0.66	−0.19
Self-discipline	3.72 ± 0.62	3.67 ± 0.67	0.44
Competence	3.36 ± 0.63	3.37 ± 0.62	−0.41
Order	3.55 ± 0.45	3.48 ± 0.51	1.00
Deliberation	3.45 ± 0.65	3.28 ± 0.63	1.74
Openness to feelings	3.34 ± 0.51	3.44 ± 0.50	−1.21
Warmth	3.47 ± 0.62	3.58 ± 0.61	−1.18
Dutifulness	3.75 ± 0.51	3.72 ± 0.52	0.33
Optimism	3.59 ± 0.69	3.15 ± 0.76	4.04**

*P < 0.05.

**P < 0.01.

*The tests used for assessment of the respective psychological variables*.

### Coping Styles

As shown in Table 4, bereaved survivors reported higher scores for avoiding problems, self-blame, and fantasy than non-bereaved survivors (*p*’s < 0.001), whereas there were no differences between the two groups regarding asking for help from others, rationalization, or solving problems.

**Table 4:** **Coping style differences between bereaved and non-bereaved survivors (*M* ± SD)**.

Factor	Bereaved survivors	Non-bereaved survivors	*t*
Avoiding problem	3.36 ± 0.92	2.92 ± 0.92	−3.23**
Help from others	3.24 ± 0.80	3.19 ± 0.78	−0.43
Self-blaming	3.11 ± 1.13	2.43 ± 1.04	−4.19**
Rationalization	3.48 ± 0.80	3.29 ± 0.71	−1.68
Fantasy	3.72 ± 0.66	3.49 ± 0.63	−2.40**
Solving problem	3.66 ± 0.68	3.55 ± 0.71	−1.01

**P < 0.01.

*The tests used for assessment of the respective psychological variables*.

### Relationships among Personality, Coping Styles, and Psychosomatic Status

As seen in Table 5, psychosomatic status scores were negatively correlated with gregariousness, competence, deliberation, and optimism (*r* = −0.51 to −0.29, *P* < 0.01) and positively correlated with fantasy and tender mindedness (*r* = 0.28–0.33, *P* < 0.01). In addition, psychosomatic status scores were negatively correlated with solving problems (*r* = −0.29, *P* < 0.05) but positively correlated with the other coping styles (*r* = 0.29–0.66, *P* < 0.01).

**Table 5:** **The correlations among personality, coping styles, and psychosomatic status**.

		Correlation with psychosomatic status	
		*r*	*P*
Personality	Gregariousness	−0.29	0.000
	Openness to fantasy	0.33	0.000
	Excitement seeking	−0.08	0.303
	Trust	−0.14	0.070
	Altruism	−0.01	0.891
	Compliance	−0.18	0.063
	Tender-mindedness	0.28	0.006
	Self-discipline	−0.14	0.060
	Competence	−0.32	0.000
	Order	0.01	0.996
	Deliberation	−0.44	0.000
	Openness to feelings	0.11	0.179
	Warmth	0.08	0.308
	Dutifulness	−0.09	0.226
	Optimism	−0.51	0.000
Copy style	Avoiding problem	0.66	0.000
	Help from others	0.29	0.000
	Self-blaming	0.58	0.000
	Rationalization	0.52	0.000
	Fantasy	0.33	0.000
	Solving problem	−0.29	0.000

*The tests used for assessment of the respective psychological variables*.

Multiple regression analysis revealed the following predictors of psychosomatic status of bereaved survivors: the optimism and openness to feelings personality characteristics and the self-blaming, avoiding problems, and rationalization coping styles (Table 6). Predictors for non-bereaved survivors were as follows: openness to fantasy, optimism, order, and trust personality characteristics and self-blaming and avoiding problems coping styles.

**Table 6:** **Multiple regression analysis among personality, coping styles, and psychosomatic status**.

Groups	Variables	*R*^2^	*B*	SE **β**	**β**	*t*-value
Bereaved survivors	Constant	0.70	20.73	6.793		3.05**
	Self-blaming		3.01	0.738	0.33	4.08**
	Avoiding problem		1.92	0.967	0.17	1.99*
	Rationalization		2.94	0.950	0.23	3.09*
	Optimism		−4.15	1.024	−0.30	−4.06**
	Openness to feelings		2.89	1.404	0.14	2.06*
Non-bereaved survivors	Constant	0.70	−1.21	9.482		−0.13
	Avoiding problem		0.28	0.059	0.39	4.78**
	Self-blaming		0.16	0.061	0.22	2.67**
	Openness to fantasy		4.52	0.998	0.27	4.53**
	Optimism		−4.54	1.094	−0.27	−4.15**
	Order		4.71	1.512	0.18	3.12**
	Trust		−3.01	1.104	−0.16	−2.73**

*P < 0.05.

**P < 0.01.

*The tests used for assessment of the respective psychological variables*.

## Discussion

The assessment of psychosomatic status demonstrated that bereaved survivors had higher scores for multiple negative emotions, such as depressed mood, loneliness, and becoming fearful easily, than non-bereaved survivors. These findings were also in line with the previous studies, which found that bereaved individuals had a more serious mental condition and more negative emotions than non-bereaved individuals (19, 28–31). Consistent with the findings of previous studies, the present study indicated that bereaved survivors may experience more serious negative emotions and poorer mental conditions than non-bereaved survivors.

In addition, the present study found that in terms of personality dimensions, bereaved individuals reported lower gregariousness, trust, and optimism than non-bereaved survivors, which was consistent with previous research (29, 32). These findings indicated that the emotion state that our measure of personality reflected among bereaved participants may be poorer than those of non-bereaved survivors (33). Based on the current exploratory analysis, the results were justifiable. We speculate that this may be due to the double impact of the earthquake and having lost relatives. After losing family members or relatives in an earthquake, bereaved survivors may experience deeper grief over the loss, exhibit more unwillingness to communicate with others, and/or may be more fearful of facing or accepting harsh realities. The interpretation above was based on research conjecture. This has to admit that generalization of the results should be cautioned. Replication study may be conducted in future. It is worth mentioning that we do not know whether the differences were temporary or permanent, or how long it would last. Future study may follow disaster survivors to see the associations of bereavement with personality traits over time. Future research also may use the more complicated statistical analysis, such as adjusting for multiple testing and moderator or mediator testing to explore the relationship among personality traits, mental health, and bereavement/non-bereavement.

This study also found that bereaved survivors had higher scores for negative coping styles, including avoiding problems, self-blame, and fantasy, than non-bereaved survivors. The present study indicated that the coping styles of bereaved survivors tended to be negative compared to those of non-bereaved survivors (34). These results were similar to other findings that negative coping styles of bereaved survivors who endured an earthquake were more closely related to negative emotions (13, 17, 35). It may be due to that their loss may have bereaved them of social support. Someone who could have provided support now was dead or the loss of a relative impacted the capacity of the survivor’s support network to provide support in a negative way. It would be particularly valuable for future studies to develop a series of interventions or psycho-educational programs to improve coping strategies in earthquake survivors, especially bereaved survivors.

The correlation analysis showed personality and coping styles were significantly related to psychosomatic status. Regression analysis revealed that optimism, self-blame, and avoiding problems were significant predictors of psychosomatic status in bereaved and non-bereaved survivors. These findings were consistent with many studies showing that positive personality characteristics were related to better psychosomatic status (36) and more negative coping styles were associated with worse psychosomatic status (16, 37). Meanwhile, the study found rationalization and openness to feelings significantly predicted psychosomatic status of bereaved survivors, while trust, openness to fantasy, and order significantly predicted the psychosomatic status of non-bereaved survivors. These findings indicated that different personality characteristics and coping styles were associated with the psychosomatic status of bereaved and non-bereaved survivors. Given that there are few empirical studies on the relationships among personality, coping, and psychosomatic state in bereaved and non-bereaved earthquake survivors (19), this study makes an important contribution to the existing literature and practice, particularly in the Chinese context.

In short, the present findings generally echoed the theoretical perspective that people experienced PTSD symptoms and negative emotions in disasters (i.e., earthquakes), and those bereaved survivors in these disasters experienced more serious PTSD symptoms and more negative emotions when compared to non-bereaved survivors (19). The findings indicate the necessity of appropriate psychological crisis interventions or psycho-educational programs, such as improving social support and coping strategies, for bereaved and non-bereaved earthquake survivors.

Several limitations exist in the current study. First, its cross-sectional design precludes inferences about causality. Second, despite steps taken to ensure the quality of data collection, factors (e.g., previous psychiatric illness) not considered may have affected data quality. Third, because of resource limitations and the severity of the environment, random sampling was not possible, so the generalization of the results may be limited. Moreover, non-random sample selection probably caused potential selection bias. Finally, this study only considered Chinese earthquake survivors, but cultural factors may affect survivors’ responses (38). For example, Chinese survivors were more likely to suppress their emotions (38) relative to Western survivors.

## Ethics Statement

This study was approved by the Human Research Ethics Committee of South China Normal University. Informed written consent was obtained from participants before the assessment. Participants were also told that they could choose not to answer any items on the questionnaires and they were free to withdraw from the study at any time during data collection.

## Author Contributions

YHX and LM contributed to the design of this research, performed the statistical analysis, drafted and revised manuscript. XC performed the statistical analsis, drafed and revised the manuscript. YQJ and RFW drafted the manuscript, polished language of the manuscript.

## Conflict of Interest Statement

The authors declare that the research was conducted in the absence of any commercial or financial relationships that could be construed as a potential conflict of interest.

## References

[1] McDermott BM Cobham VE Berry H Stallman HM. Vulnerability factors for disaster-induced child post-traumatic stress disorder: the case for low family resilience and previous mental illness. Aust N Z J Psychiatry (2010) 44:384–9. 10.3109/00048670903489916 20307172

[2] Wickrama KA Kaspar V. Family context of mental health risk in tsunami-exposed adolescents: findings from a pilot study in Sri Lanka. Soc Sci Med (2007) 64:713–23. 10.1016/j.socscimed.2006.09.031 17084953

[3] Vigil JM Geary DC. A preliminary investigation of family coping styles and psychological well-being among adolescent survivors of hurricane Katrina. J Fam Psychol (2008) 12:176–80. 10.1037/0893-3200.22.1.176 18266546

[4] Rowe CL La Greca AM Alexandersson A. Family and individual factors associated with substance involvement and PTS symptoms among adolescents in greater New Orleans after hurricane Katrina. J Consult Clin Psychol (2010) 78:806–17. 10.1037/a0020808 20919759

[5] Armenian HK Morikawa M Melkonian AK Hovanesian A Akiskal K Akiskal HS. Risk factors for depression in the survivors of the 1988 earthquake in Armenia. J Urban Health (2002) 79(3):373–82. 10.1093/jurban/79.3.373 12200506 PMC3456783

[6] Bonanno GA Galea S Bucciarelli A Vlahov D. What predicts psychological resilience after disaster? The role of demographics, resources, and life stress. J Consult Clin Psychol (2007) 75(5):671. 10.1037/0022-006X.75.5.671 17907849

[7] Salcioglu E Basoglu M Livanou M. Post-traumatic stress disorder and comorbid depression among survivors of the 1999 earthquake in Turkey. Disasters (2007) 31(2):115–29. 10.1111/j.1467-7717.2007.01000.x 17461919

[8] O’Toole BE Marshall RP Schureck RJ Dobson M. Risk factors for posttraumatic stress disorder in Australian Vietnam veterans. Aust N Z J Psychiatry (1998) 32:21–31. 10.3109/00048679809062702 9565180

[9] Fauerbach JA Lawrence JW Schmidt CW Personality predictors of injury related posttraumatic stress disorder. J Nerv Mental Dis (2000) 188:510–7. 10.1097/00005053-200008000-00006 10972570

[10] Zhang Y Kong FC Wang L Chen H Gao X Mental health and coping styles of children and adolescent survivors one year after the 2008 Chinese earthquake. Child Youth Serv Rev (2010) 32:1403–9. 10.1016/j.childyouth.2010.06.009

[11] Weiten W Psychology Themes and Variations. 6th ed Belmont, CA: Wadsworth, Thomson Learning (2004). p. 127–36.

[12] Asarnow J Glynn S Pynoos RS Nahum J Guthrie D When the earth stops shaking: earthquake sequelae among children diagnosed for pre-earthquake psychopathology. J Am Acad Child Adolesc Psychiatry (1999) 38:1016–23. 10.1097/00004583-199908000-00018 10434494

[13] Carr VJ Lewin TJ Webster RA Hazell PL Kenardy JA. Psychosocial sequelae of the 1989 Newcastle earthquake: I. Community disaster experiences and psychological morbidity 6 months post-disaster. Psychol Med (1995) 25:539–55. 10.1017/S0033291700033468 7480435

[14] Karanci NA Alkan N Aksit B Sucuoglu H Balta E Gender differences in psychological distress, coping, social support and related variables following the 1995 Dinar (Turkey) earthquake. North Am J Psychol (1999) 1(2):189–204.

[15] Schnider KR Elhai JD Gray MJ Coping style use predicts posttraumatic stress and complicated grief symptom severity among college students reporting a traumatic loss. J Couns Psychol (2007) 54(3):344 10.1037/0022-0167.54.3.344

[16] Dong HJ Gu JH Zou QJ Yang YB On psychological influence and individual coping with vital emergent events a case of India Ocean tsunami. J Nat Disasters (2006) 15:88–91.

[17] Pina AA Villalta IK Ortiz CD Gottschall AC Costa NM. Social support, discrimination, and coping as predictors of posttraumatic stress reactions in youth survivors of hurricane Katrina. J Clin Child Adolesc Psychol (2008) 37:564–74. 10.1080/15374410802148228 18645747

[18] Norris FH Friedman MJ Watson P 60,000 Disaster victims speak: part 1. An empirical review of the empirical literature. J Psychiatry (2002) 65:207–39. 10.1521/psyc.65.3.207.20173 12405079

[19] Chan CL Wang CW Ho AH Qu ZY Wang XY. Symptoms of posttraumatic stress disorder and depression among bereaved and non-bereaved survivors following the 2008 Sichuan earthquake. J Anxiety Disord (2012) 26:673–9. 10.1016/j.janxdis.2012.05.002 22721751

[20] Weisaeth TH. Psychiatric disorders among disaster bereaved: an interview study of individuals directly or not directly exposed to the 2004 tsunami. Depress Anxiety (2009) 26:1127–33. 10.1002/da.20625 19998267

[21] Yuan Y Mao W-J Yang D-H Ran M-S Kong D Zhang T Comparison of the PTSD symptoms, depression and anxiety between bereaved and non-bereaved survivors after Wenchuan earthquake. Chin J Behav Med Brain Sci (2009) 18(12):1109–11.

[22] Costa PT McCrae RR Revised NEO Personality Inventory (NEO PI-R) and NEP Five-Factor Inventory (NEO-FFI): Professional Manual. Lutz, FL: Psychological Assessment Resources (1992).

[23] Huang F Higher order factors of the big five: social relations model. Psychol Explore (2014) 34(3):236–42.

[24] Cui J Deng G Dong W Correlation between teenagers’ perceived social support and their personality four years after Wenchuan earthquake. Chin J Health Psychol (2014) 22(10):1551–53. 10.13342/j.cnki.cjhp.2014.10.046

[25] Wang JL Jackson LA Zhang DJ Su ZQ The relationships among the big five personality factors, self-esteem, narcissism, and sensation-seeking to Chinese University students’ uses of social networking sites. Comput Hum Behav (2012) 28(6):2313–19. 10.1016/j.chb.2012.07.001

[26] Zheng L Goldberg LR Zheng Y Zhao Y Tang Y Liu L Reliability and concurrent validation of the ipip big-five factor markers in China: consistencies in factor structure between internet-obtained heterosexual and homosexual samples. Pers Individ Differ (2008) 45(7):649–54. 10.1016/j.paid.2008.07.009 PMC2851096 20383283

[27] Wo JZ Liu HJ A study on the features of emotion of primary pupils. Chin J Clin Psychol (2003) 11(2):102–6.

[28] Barnet LA The Long-Term Effects of Parental Functioning and the Family Environment on Child Psychopathology in Bereaved 9/11 Families (Order No. 3537853) at ProQuest Dissertations and Theses A&I: The Humanities and Social Sciences Collection (1324166854) (2012). Available from: http://search.proquest.com/docview/1324166854?accountid=13833

[29] Cao X Jiang X Li X Lo MJ Li R. Family functioning and its predictors among disaster bereaved individuals in China: eighteen months after the Wenchuan earthquake. PLoS One (2013) 8(4):e60738. 10.1371/journal.pone.0060738 23573280 PMC3616101

[30] Chen J Yang XG Wang JG Preliminary study on psychological resilience of survivor after a catastrophe: a case study of 1976 Tangshan earthquake. J Nat Disasters (2008) 17:86–91.

[31] Zhang B Wang XY Sun HX Long-term effects of Tangshan earthquake on psychosomatic health of mankind. Chin Ment Health J (1998) 12:200–2.

[32] Cohen NL Grief and Trauma: An Empirical Investigation of the Construct Overlap and the Psychological and Physical Functioning of Bereaved Individuals with and without Complicated Grief (Order No. NR11561) at ProQuest Psychology Journals (305367726) (2005). Available from: http://search.proquest.com/docview/305367726?accountid=13833

[33] Milojev P Osborne D Sibley CG Personality resilience following a natural disaster. Soc Psychol Personal Sci (2014) 5(7):760–8. 10.1177/1948550614528545

[34] Wang XD Wang XL Ma H Mental health assessment scale. Chin Ment Health J (1999) 29:312–45.

[35] Kuo CJ Tang HS Tsay CJ Lin SK Hu WH Chen CC Prevalence of psychiatric disorders among bereaved survivors of a disastrous earthquake in Taiwan. Psychiatr Serv (2014) 54(2):249–51. 10.1176/appi.ps.54.2.249 12556609

[36] Zhang LY Shi JN Li YG Influence of disastrous incident to somatic and mental health of the relatives of the victims and the intervention study. Chin J Clin Psychol (2008) 16:89–91.

[37] Xiang H Yan LJ Zhang HW Correlation between coping style and mental health of high school students. Health Med Res Pract High Inst (2008) 15:11–4.

[38] Butler EA Lee TL Gross JJ. Emotion regulation and culture: are the social consequences of emotion suppression culture-specific? Emotion (2007) 7(1):30. 10.1037/1528-3542.7.1.30 17352561

